# Music therapy in Huntington’s disease: a protocol for a multi-center randomized controlled trial

**DOI:** 10.1186/s40359-016-0146-z

**Published:** 2016-07-26

**Authors:** Monique van Bruggen-Rufi, Annemieke Vink, Wilco Achterberg, Raymund Roos

**Affiliations:** 1 Department of Neurology, Leiden University Medical Center, Leiden, Netherlands; 2 ArtEZ School of Music, Enschede, Netherlands; 3 Atlant Care Group, Apeldoorn, Netherlands; 4 Department of Public Health and Primary Care, Leiden University Medical Center, Leiden, Netherlands; 5 Topaz Huntington Center Overduin, Katwijk, Netherlands; 6 KenVaK, Zuyd University, Heerlen, Netherlands

**Keywords:** Music therapy, Huntington’s disease, Communicative and expressive skills, Behavioral problems, Quality of life, Randomized controlled trial

## Abstract

**Background:**

Huntington’s disease is a progressive, neurodegenerative disease with autosomal dominant inheritance, characterized by motor disturbances, cognitive decline and behavioral and psychological symptoms. Since there is no cure, all treatment is aimed at improving quality of life. Music therapy is a non-pharmacological intervention, aiming to improve the quality of life, but its use and efficacy in patients with Huntington’s disease has hardly been studied.

In this article, a protocol is described to study the effects of music therapy in comparison with a control intervention to improve quality of life through stimulating expressive and communicative skills. By targeting these skills we assume that the social-cognitive functioning will improve, leading to a reduction in behavioral problems, resulting in an overall improvement of the quality of life in patients with Huntington’s disease.

**Methods/Design:**

The study is designed as a multi-center single-blind randomised controlled intervention trial. Sixty patients will be randomised using centre-stratified block-permuted randomisation. Patients will be recruited from four long-term care facilities specialized in Huntington’s disease-care in The Netherlands.

The outcome measure to assess changes in expressive and communication skills is the Behaviour Observation Scale Huntington and changes in behavior will be assessed by the Problem Behaviour Assesment-short version and by the BOSH. Measurements take place at baseline, then 8, 16 (end of intervention) and 12 weeks after the last intervention (follow-up).

**Discussion:**

This randomized controlled study will provide greater insight into the effectiveness of music therapy on activities of daily living, social-cognitive functioning and behavior problems by improving expressive and communication skills, thus leading to a better quality of life for patients with Huntington’s disease.

**Trial registration:**

Netherlands Trial Register: NTR4904, registration date Nov. 15, 2014.

## Background

Huntington’s Disease (HD) is a progressive, neurodegenerative disease with autosomal dominant inheritance, caused by an elongated CAG repeat on chromosome 4 [1]. HD is characterized by motor disturbances, cognitive decline and behavioral and psychosocial symptoms. The cognitive disorder in HD effects a broad variety of skills, including learning and memory, perceptual skills, executive efficiency and language [2]. Cognitive and behavioral changes are the most debilitating aspects of the disease and place the greatest burden on the patient as well as on their families and caregivers, and are often the main reason for institutionalization [3, 4]. Affective (mood) disorders (e.g. depression, anxiety, apathy, irritability) and behavioral problems (e.g. obsessive compulsive behavior and aggression) are frequent symptoms [5].

Verbal communication is often affected, due to motor impairment of speech. In addition, as a result of the cognitive decline, word retrieval is often impaired, although the knowledge of vocabulary is retained. As the disease progresses, the language content can no longer be processed properly and in the later stages of the disease, a patient with HD might not be able to communicate adequately [1].

As a result of the communication problems, patients are no longer able to express themselves. Revealing the patients’ unmet needs is, therefore, hindered and this might lead to irritability, impulsive and unwanted behavior (frequently signs in HD), causing much distress for patients and caregivers. Because of the behavioral symptoms, cognitive decline and the inability to express oneself, psychosocial problems develop [6]. Psychosocial stressors may include feelings of sadness and anxiety about the cognitive and physical decline, and about changes in social roles [7]. The gradual deterioration in communication skills, in combination with the behavioral problems in patients with HD, contributes to a decrease of functional health and a progressive inability to participate in various life situations, leading to loss of quality of life [8].

Since there is no cure for HD, the aim of all therapy, both pharmacological and non-pharmacological, is to improve quality of life [9]. One form of non-pharmacological intervention is music therapy.

Music therapy uses music experiences and patient-therapist relationships in order to effect therapeutic change [10]. Over the past decades, music therapy has been developed for patients with neurodegenerative diseases [11, 12]; its efficacy is shown in Table 1.

**Table 1:** Benefits of music therapy for the neurodegenerative diseases

Benefits	Reference	Population
Improving and regulating emotional wellbeing	[13, 31]	Dementia, Huntington
Increasing social response	[13, 31]	Dementia, Huntington
Decreasing agitation	[13]	Dementia
Recalling life experiences	[14, 15, 31]	Dementia, Huntington
(re)establishing contact	[14, 15, 31]	Dementia, Huntington
Improving communication skills	[11, 12, 16, 31]	Dementia, Parkinson, Huntington, multiple disabilities
Reducing behavior and psychological symptoms	[17–20, 31]	Dementia
Improving gait	[9]	Parkinson

There is evidence that music therapy influences emotional well-being positively and that participation in music therapy increases social response in people with dementia [13]. It can decrease agitation and has a positive effect on enhancing communication and emotional well-being. Music therapy enables the recall of life experiences and the experience of pleasant emotions. Through music, contact can be established, especially as language deteriorates during the later stage of the dementing process [14, 15].

In a comprehensive literature search, Lee and McFerran [16] conclude that the non-verbal communication skills in individuals with profound and multiple disabilities improve as a result of song-choice during music therapy sessions. The results of their study support the belief that, through music therapy, the ability to express oneself will improve, contributing to improvement in the quality of life.

Furthermore, in a literature review, Patel et al. [17] demonstrate the effectiveness of MT in reducing behavioral and psychological symptoms of dementia. Several other meta-analyses and literature reviews have concluded that music therapy decreases symptoms related to behavioral problems in dementia [18–20].

On the basis of the above-mentioned literature, the assumption can be made that music therapy might be beneficial to patients with HD. Although knowledge on the use and efficacy of music therapy in HD is limited, it has been suggested that it can improve the communication skills of people with HD [21]. By improving the expressive and communication skills through music therapy (which will be assessed by the BOSH) we hypothesize that behavioral problems will decrease (assessed by the BOSH and the PBA-s), leading to an overall improvement of quality of life.

To test this hypothesis, we wrote a study protocol to answer the following questions:

1. Does music therapy improve expressive and communicative skills in people with HD?
2. Does music therapy reduce behavioral problems in patients with HD?
3. Does music therapy improve the quality of life of patients with HD?

## Methods

### Study design

**Fig. 1:**
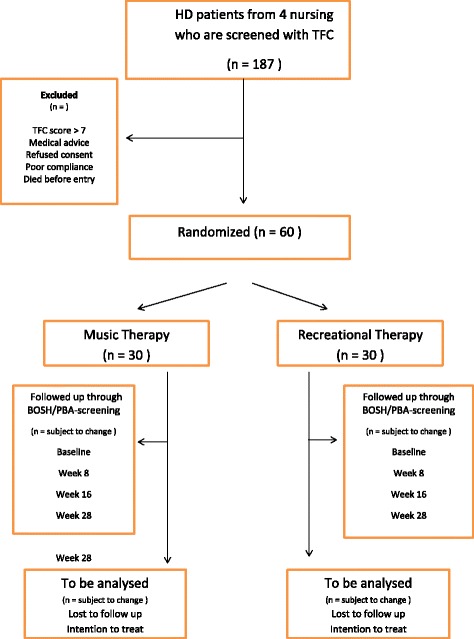
Flowchart

The study is a multi-centre, single-blind, randomised controlled intervention trial with two parallel arms. An overview of the study design is shown in a flow chart (see Fig. 1). It is single-blinded; the researcher who analyses all the scores is unaware of the allocation of the patients throughout the study. Also, the persons performing the baseline-assessments will be kept unaware of the allocation of the participants they are testing. Sixty patients (see sample size calculation below) will be randomised using centre-stratified, block-permuted randomisation following the procedure as described below. Two random groups will be created. The experimental group will be offered a music therapy program according to a structured protocol (see below), and the control group will participate in recreational day activities, following the same protocol as the experimental group. In addition, both groups will receive regular treatment (standard care, treatment as usual). Participants from both the experimental and the control groups will not be allowed to receive music therapy outside the study.

### Procedure

#### Recruitment

Patients will be recruited from four long-term care facilities (nursing homes) in The Netherlands, all specialized in HD, with one or more units exclusively for HD-patients. Recruitment will be primarily done by the nursing staff and the responsible physician.

### Inclusion and exclusion criteria

Eligible patients must meet the following inclusion criteria:

- Age older than 18 years
- Clinically and genetically confirmed Huntington’s Disease (CAG ≥ 36 repeats)
- Total Functional Capacity (TFC) ≤7

Exclusion criteria:

- patients who suffer from other neurological disorders
- patients with poor comprehension of the Dutch language
- patients who have received music therapy in the past 3 months
- patients with a hearing impairment

Patients will be included irrespective of medication status and will be allowed to continue medication during the study. However, any change in medication should be avoided and carefully registered.

### Informed consent

Informed consent will be obtained by the nursing staff from patients who are cognitively able to understand the possible risks and benefits of the study. Proxy consent will be obtained from next of kin, or a legal guardian, if the patient is not able to give an informed consent.

### Baseline assessments

Clinical and demographic variables such as gender, age, (changes in) medication, number of CAG-repeats and (estimated) age at onset will be gathered for each participant. The stage of Huntington’s Disease will be assessed using the Total Functional Capacity (TFC) [22]. The TFC, part of the Unified Huntington Disease Rating Scale, consists of 13 points and contains 5 domains: work, finances, domestic chores, activities of daily living and care requirements [22]. As the disease progresses, the TFC-score will drop, giving clinicians a good indication about the functional stage of the disease. A score of 7 or lower indicates that the patient is in the mid/advanced stage, and so impaired in his functional capacities that assistance with self-care is needed [22]. Most of the patients that reside in the four long term care facilities that participate in the study have a TFC-score of 7 or lower. To ensure a homogeneous group, patients with a TFC score of > 7 will be excluded from participating in the research The TFC will be administered by the elderly care physician who is responsible for the patient.

The Mini Mental State Examination (MMSE) will be used to assess the cognitive function of the patients at baseline. A score of 23 or lower out of 30 indicates cognitive impairment [23]. The MMSE will be administered by the psychologist or the psychology assistant.

### Randomisation

In order to minimize the impact of any between-center differences on the trial results, the participating centers will be stratified in the randomization process. Participants will be randomly assigned to either control or experimental group with a 1:1 allocation. To generate the random allocation sequence we use the method of center-stratified, block-permuted randomization [24]. The block size will vary. To diminish the chance of predictability, the date of signing the informed consent will determine the sequence in which the patients will be randomized. Not until all baseline measurements have been completed, the randomization code will be released to ensure allocation concealment.

Randomization will be conducted without any influence of the principal investigators, raters or therapists.

### Intervention

All participants (in both the experimental (intervention) group and the control group) will continue to receive treatment as usual. The intervention group will receive music therapy (MT-group). The control group will receive recreational therapy. The number of sessions (16) is equal in both groups, as are the day of the week and the time of the day at which the sessions will take place. Patients in both groups will participate in group interventions with three to five participants.

It must be noted that each of the four participating facilities have a different music therapist and recreational therapist attached to the research. However, all participating therapist have been instructed thoroughly to follow the same protocol. The reports that the therapist will fill out after each session will be used to monitor whether they have followed the instructions. See "compliance with treatment".

### Intervention group

The music therapists committed to the study are professionally trained and have been specifically informed about the clinical method and its theoretical basis.

The main goal of the music therapy intervention is to enhance communication skills by way of encouraging and stimulating the patients in interaction. The music therapy approach applied in this study is focused on encouraging and engaging patients in expressive musical interaction. The role of the therapist is to use musical parameters and interventions to stimulate expressive and communicative skills. The degree of verbal reflection may vary; the therapist will, however, encourage the participants to express themselves. The therapeutic process is based on the mutual construction of meaning of emerging thoughts, images, emotional content and expressive qualities that often originate from the musical experience [10].

A treatment guide specifies the procedures. It outlines the setting, goals and basic principles of the intervention; Table 1 (the benefits of music therapy for neurodegenerative diseases) is used as guideline. The available music therapy techniques to target the set goals are derived from the protocol “music therapy for Huntington’s patients on improving and stimulating communication and self-expression” [25]. However, the guidelines are to be administered flexibly according to the patient’s state of mind and his needs at that very moment. The clinical expertise of the therapist will be the guide, providing the therapist with enough “space” for flexible adaptation within the treatment guide. Also, the patients music preference, especially because most of the treatment involves receptive music therapy, is very important. This is the reason why the protocol allows and encourages the music therapist to adjust their treatment by way of “tailor made” sessions, providing each of the participants with his or her music preference.

The process used in each session is standard while the content is flexible. The intervention will be provided at the same time of the same day of the week by a formally trained, experienced music therapist. The sessions will take place once weekly with a total of 16 sessions, lasting 45 min. They will be standardized without limiting the music therapists in their interactions. The intervention itself, however, will be (partly) applied according to a protocol. Each session starts with the same welcome song/musical piece and ends with the same farewell song/musical piece. In doing so, the participants become familiar with the start and the end of each session. In between these two songs/musical pieces, the music therapist adjusts the level of each intervention to individual capacities. After the welcome song, the music therapy sessions may be varied: the music experiences can range from listening to music to playing or singing songs to free improvisation. therapist has the freedom to determine what works best at that very moment for that specific patient. The participants will listen to music selected, sung or played by the therapist. Active participation in music activities by singing or playing a musical instrument will be stimulated as much as possible. The music will be selected by the music therapist to incite expressive and communication skills and to reduce agitation, based on musical parameters, such as rhythm, melody, harmony, dynamics, timbre. After each song/musical intervention, the therapist will encourage and stimulate the participants to reflect verbally on the music [25].

Besides the music therapy intervention during the whole study, participants are not allowed to receive additional individual music therapy.

All participants are allowed to leave the session at all times.

### Control group

All activities will be provided by professionally trained recreational therapists who have been specifically informed about the study.

In the control group, recreational day activities will be offered under exactly the same circumstances as the music therapy sessions: a total of 16 weekly sessions, each lasting 45 min, every week at exactly the same time as the music therapy intervention. As in the intervention group, a treatment guide specifies the treatment procedures for the control group. In this guide, the setting and general goals are outlined. The main goal of the recreational activities is to enhance communication skills by way of encouraging and stimulating the patients in interaction.

The activities vary from reading the newspaper, cooking, arts-and-crafts/handwork or puzzles/games. Musical activities, such as singing along or watching a music-video are not allowed, nor will background-music be played. The recreational therapist is well instructed about and fully aware of this restriction. Besides that, during the whole study, participants from the control group are not allowed to receive music therapy. Both the physician who is responsible for the referrals and the music therapists are fully aware of this limitation. Participation in in regular musical activities however (such as watching a music video or attending a music-performance which takes place on the ward occasionally) is allowed. These activities are not considered to be music therapy and are open to all patients that reside in the institution, regardless of participation in the study. Also, listening to music in the privacy of their own room is allowed for all participants.

All participants are allowed to leave the session at all times.

The music therapy and the recreational day activities will be provided in separate rooms, away from the ward. Participants will be taken to the music therapy room or the activity room by the nursing staff. The music therapist and the recreational therapist make sure that they can start the moment all participants are in the room. After the session, the participants will be taken back to the ward by the nursing staff. The therapists will never leave a participant in the room unattended.

After each session, a short report of the activities will be written by both therapists, including an evaluation of each patient. Since a self-report from the patient himself is not feasible, the reports written by the therapists will be used for evaluation purposes and treatment fidelity.

### Compliance with treatment

In each participating center a monitor will be identified, to be responsible for adherence to study protocol (including data collection).

Furthermore, after each session, both therapists will document significant events, notable behaviors, and interventions applied. These reports will enable us to determine whether the treatment has been conducted as intended. These reports will provide insight into the patient’s state of mind at the time of the intervention. This information can help us explain why certain measurements might be significantly different compared to others in the same patient. Self-reports (the patients’ own perception) might not be feasible, due to the rapid decline of cognitive skills (see rationale), Furthermore, this information will also be used to evaluate the process of the study.

### Outcome measures

The primary outcome measure, communication skills, will be assessed by the social-cognitive domain of the Behaviour Observation Scale Huntington (BOSH) [26]. The secondary outcome measure, behavior, will be assessed by both the Problem Behaviours Assessment-short version [27, 28] and the third domain (mental rigidity and aggression) of the BOSH (mental rigidity and aggression).

The BOSH contains 32 items in 3 subscales: 1) activities of daily living (ADL) (5 items), 2) social-cognitive functioning (15 items), and 3) mental rigidity and aggression (12 items). Each item is assessed on a 4-point scale. The scores from the first subscale (ADL) range from 1 (self-supporting) to 4 (nursing required). The sum score from the first scale may range from 5 to 20. The scores from the second subscale (social-cognitive functioning) range from 1 (unaffected) to 4 (contact no longer possible); the sum score may range from 15 to 60. Finally, the scores from the third subscale (mental rigidity and aggression) range from 1 (never) to 4 (always); the sum score may range from 12 to 48. Intra- and interrater reliabilities are respectively 0.83 and 0.95 [26].

Assessment of the BOSH will take place within a week before the first intervention (baseline assessment), and will be repeated within a week after the 8th and again within a week after the 16th intervention. 12 weeks after the last intervention, the BOSH will be repeated.

The BOSH takes 15 min and will be administered by nursing staff in charge of the daily care of the patients. Blinding the BOSH-assessors might not be feasible as the nursing staff will have to transport the patients to and from the therapy-rooms. Also in daily life, the chance of the patient divulging his or her allocation towards the nursing staff is big. However, in the process-evaluation following the study all the assessors will be asked whether they were aware of the allocation of the patients.

Since the BOSH is a commonly known assessment scale in the participating HD nursing homes in The Netherlands, no formal training will be carried out prior to the start of the study unless the particular nursing home has no experience with the BOSH.

Behavioral problems will be assessed with the Problem Behaviours Assessment-short (PBA-s) version [27, 28]. This is a 5-point rating scale, using the scores 0 (absent) to 4 (severe). The PBA-s is a 11-item semi-structured interview and assesses behavioural problems in the 4 weeks prior to the interview. The sum score may range from 0 to 40. In addition, there is a subscale for severity and one for frequency. The PBA-s is a validated and reliable measurement-tool: the interrater reliability is 0.74 for severity and 0.76 for frequency scores (mean kappa), and 0.94 for severity and 0.92 for frequency scores (weighted kappa) [29]. The respondents of this face to face semi-structured interview are the patient and a knowledgeable informant (spouse or caregiver) together.

Assessment of the PBA-s will take place following the same time-schedule as the BOSH-assessments. The PBA-s will be scored by independent, formerly trained assessors who will be blinded to group allocation of the patients. After the last assessment, the assessor will be asked whether or not he inadvertently found out about the patient’s allocation in order to verify the success of the blinding. Where possible, subjects are interviewed in the presence of a knowledgeable informant (primary caregiver).

After consulting a member of the PBA-workgroup we decided to adjust the 4-week retrospective view of the PBA-s to 1 week, due to the short time-frame in which it is administered (every 8 weeks).

### Sample size

The minimal clinically important change or difference (MCIC/MCID) in the scale serves as important input for the sample size calculations. Unfortunately, data about these scale-characteristics are unavailable. We anticipate that the population in the present study will differ little from the population previously used in the study by Timman et al. [26] from which we derived data on means and standard deviations for the subscales of the BOSH. If we assume that the values in the control group will change little over the course of the study, whereas those in the experimental group will improve by 25 %, and if we further assume an α of 0.05 and a β of 0.20, then the following sample size would be required:

- ADL: original mean ± SD = 2.25 ± 0.88; improvement of 25 % will result in a mean of 1.69 and if we conservatively estimate a reduction in SD to 0.75, we would require an N of 30 per group for the ADL subscale (for an effect size of 0.55 (moderate));
- Social-Cognitive: original mean ± SD = 2.10 ± 0.79; improvement of 25 % will result in a mean of 1.58 and if we conservatively estimate a reduction in SD to 0.75, we would also require an N of 30 per group for this subscale (for an effect size of 0.52 (moderate)).

### Data management and confidentiality

Participant files will be stored in locked cabinets with limited access. Participating centers will only have access to their own center’s data. Data will be entered into SPSS by an independent research assistant. After the trial the principal investigators and the statistician will have access to the data set.

### Statistical analysis

The primary outcome measures of this study are the differences in total and subscale-scores of the ADL and Social-Cognitive subscales of the BOSH and the PBA-s between groups (control versus MT) when baseline differences are taken into account.

Results will be analyzed on an intention-to-treat basis.

A mixed model analysis with repeated measures will be used to analyze the differential effects of music therapy versus recreational activities on the four GOSH and PBA-s scores (week 0, week 8 and week 16, and a follow-up score in week 28). The assessment number, ranging from one to four, will be used as the time variable of the repeated measures.

As stratified randomization often leads to correlation between treatment arms, it is necessary to adjust for the stratification factors in the analysis to obtain correct confidence intervals and p-values. By doing so, we maintain the type I error rate at its nominal level (usually set at 5 %), and avoid a reduction in power [30].

## Discussion

Music therapy is a promising non-pharmacological intervention. Through stimulation of the expressive and communicative skills, it is hypothesized that this therapy will result in improving activities of daily life and social-cognitive functioning as well as reducing behavioral problems, thus leading to an improvement in the quality of life.

In general, music therapy can be offered either individually or in group sessions, with other patients or with family members. The sessions can be “tailor-made” for the patient and his needs.

In this study, patients in both the intervention- and the control group will participate in group interventions with three to five participants. The decision to choose group-interventions rather than individual interventions is based on the assumption that the interaction between group-members will stimulate communication [31]. In this article, Magee concludes that music therapy is recommended in the middle and the advanced stages of HD. Group sessions allow the patient the time needed for delayed responses, allowing natural “time out” from responses which require attention or concentration. The number of participants in each group has to be determined carefully. In a systematic literature review, Ing-Randolph et al. [32] suggest that group size matters with music interventions addressing certain stages of dementia-associated anxiety. Although Yalom [33] states that a group consisting of fewer than five members results in a decrease in member interaction, this is not the case when working with HD-patients where smaller groups are preferable [31].

Based on earlier RCTs and a meta-analysis of the dose-effect relationship of music therapy in different settings and populations, we expect a treatment duration of 16 weekly sessions, each session lasting 60 min, to be sufficient for detectable developments [13, 14].

The decision to apply the BOSH as the primary outcome measures instead of a QoL-scale requires some clarification; in our opinion, no validated QoL-scale (generic nor disease-specific) is sufficiently sensitive to be used in the very late stage of the disease.

The BOSH was developed to provide an observational instrument for monitoring the behavioural aspects of the patient in later stages. Expressive and communicative skills are specifically measured within the social-cognitive functioning-subscale. In combination with its 2 other subscales (ADL and mental rigidity), we believe that the scale is so broad that it covers most of the QoL-domains. In the present study, we are planning to analyze the total score of the BOSH.

## Conclusion

This article outlines the study protocol of a randomized controlled trial providing insight into the effects of a structured group music therapy intervention for patients with Huntington’s disease. More specifically, the effects of music therapy on improving quality of life through specifically targeting the expressive and communicative skills, in comparison with an active control intervention, will be tested.

Conclusions that will emerge from this study are expected to contribute to evidence-based treatment for Huntington’s Disease patients who experience deterioration of expressive and communicative skills, leading to a reduction in behaviour problems, and resulting in an overall improvement in quality of life.

### Nature and extent of the burden and risks associated with participation, benefit and group relatedness

It is not expected that the intervention will be a serious burden for or risk to the participants, nor are physical or physiological discomforts associated with this study.

### Reporting of study results

The study results will be disseminated in the following ways:

- A final report
- Formal publications in peer reviewed high impact international journal
- Presentations in different international conferences
- Dissertation and public defense

### Protocol amendments

Any modifications to or administrative changes or clarifications of the protocol that have no effect on the way the study is to be conducted will be reported to the Dutch Trial Register (NTR 4904).

## Abbreviations

BOSH, Behavioural Observation Scale for Huntington; HD, Huntington’s disease; LTCF, long term care facility; PBA-s, Problem Behaviour Assessment-short version; QoL, quality of life
